# Smart Indigenous Youth: The Smart Platform Policy Solution for Systems Integration to Address Indigenous Youth Mental Health

**DOI:** 10.2196/21155

**Published:** 2020-09-25

**Authors:** Tarun Reddy Katapally

**Affiliations:** 1 Johnson Shoyama Graduate School of Public Policy University of Regina Regina, SK Canada; 2 Community Health and Epidemiology College of Medicine University of Saskatchewan Saskatoon, SK Canada

**Keywords:** Indigenous youth, mental health, school policies, health policy, digital health interventions, mHealth, systems integration, land-based learning, physical activity

## Abstract

Indigenous youth mental health is an urgent public health issue, which cannot be addressed with a one-size-fits-all approach. The success of health policies in Indigenous communities is dependent on bottom-up, culturally appropriate, and strengths-based prevention strategies. In order to maximize the effectiveness of these strategies, they need to be embedded in replicable and contextually relevant mechanisms such as school curricula across multiple communities. Moreover, to engage youth in the twenty-first century, especially in rural and remote areas, it is imperative to leverage ubiquitous mobile tools that empower Indigenous youth and facilitate novel Two-Eyed Seeing solutions. Smart Indigenous Youth is a 5-year community trial, which aims to improve Indigenous youth mental health by embedding a culturally appropriate digital health initiative into school curricula in rural and remote Indigenous communities in Canada. This policy analysis explores the benefits of such upstream initiatives. More importantly, this article describes evidence-based strategies to overcome barriers to implementation through the integration of citizen science and community-based participatory research action.

## Indigenous Youth Mental Health: A Historical and Cultural Perspective

According to the World Health Organization, suicide is the second leading cause of mortality among individuals aged between 15 to 29 years [1].  Although suicide rates vary between and within countries across various subpopulations, evidence clearly indicates that youth—especially Indigenous youth in settler nations such as Australia, Canada, and the United States—have significantly higher rates of mental illness and suicide [2]. The United Nations identifies Indigenous Peoples as inheritors and practitioners of unique cultures and ways of relating to people and the environment, and state that they are arguably among the most disadvantaged and vulnerable groups of people in the world [3].

Indigenous Peoples’ culture, language, subsistence, and ways of life have been adversely affected by a history of colonization and forced assimilation [4,5]. The resultant intergenerational trauma continues to influence the health status of Indigenous communities [6-8]. This history of dispossession is particularly challenging for Indigenous youth due to a loss of identity, which is reflected in significant health disparities, high suicide rates, and poverty [9].

Although Indigenous Peoples were historically a healthy population [10,11], inter-generational inequities have resulted in poor holistic health [12], which encapsulates physical, mental, social/emotional, and spiritual/cultural aspects. As Indigenous Knowledge emphasizes that these varied aspects of holistic health are interdependent and interrelated, an imbalance across the spectrum of holistic health is detrimental to Indigenous Peoples [11,13].

With respect to youth mental health challenges, although there are indications of significant differences in prevalence across Indigenous communities [14], a common pattern is the dearth of Indigenous Knowledge use in facilitating youth mental health. This is disconcerting because evidence indicates that Indigenous Peoples possess the knowledge to enable culturally rich environments for youth to thrive. More importantly, while Indigenous Knowledge can be transferred across communities to enable Indigenous Ways of Knowing so that struggling communities can benefit from the success of thriving communities, this approach is rarely incorporated into policy or program decisions [14].

The push for preventive mental health policies and programs across settler nations is commendable, yet there is a risk of taking a one-size-fits-all approach in scalable interventions that are focused on top-down implementation rather than bottom-up, culturally appropriate, and strengths-based prevention strategies [15]. A potential key to the success of these strategies is the transfer of Indigenous Knowledge across communities by ensuring cultural connectedness that is specific to each community [14].

Culture incorporates spirituality, identity (individual, family, community, and nation), and traditions [16]. Strong cultural identity and cultural connectedness have been associated with better health, higher self-esteem, positive mental health, and lower rates of binge drinking [16-21]. Cultural connectedness can also act as a coping mechanism to deal with historical trauma, thus increasing personal and community health [21].

With such strong evidence of culture playing a role in holistic health, there is a need to normalize culturally appropriate policies and programs to facilitate Indigenous youth mental health [22]. However, these approaches need to be embedded in replicable and contextually appropriate mechanisms such as school curricula across multiple communities to ensure the exchange of Indigenous Knowledge [14]. In order to engage the youth of the twenty-first century, especially in rural and remote Indigenous communities, it is also imperative to leverage ubiquitous mobile tools for digital health interventions to not only address access gaps, but also to empower Indigenous youth. This approach will facilitate innovative Two-Eyed Seeing solutions [23-25], which incorporate Western digital epidemiological methods with Indigenous Knowledge.

## Smart Indigenous Youth: An Innovative Systems Integration Policy Solution

Smart Indigenous Youth embeds a land-based, culturally appropriate, active living digital health initiative into school curricula to promote mental health, minimize substance abuse, and prevent suicide among Indigenous youth (13-18 years old) in rural and remote areas of the Canadian province of Saskatchewan. In implementing Smart Indigenous Youth, we are integrating services supported by the Saskatchewan ministries of health, education, and sport, thus enabling a policy of systems integration. The initiative uses the Smart Platform [26,27], which is the first citizen science and digital epidemiological platform for ethical population health surveillance, integrated knowledge translation, and policy and real-time interventions.

**Figure 1:**
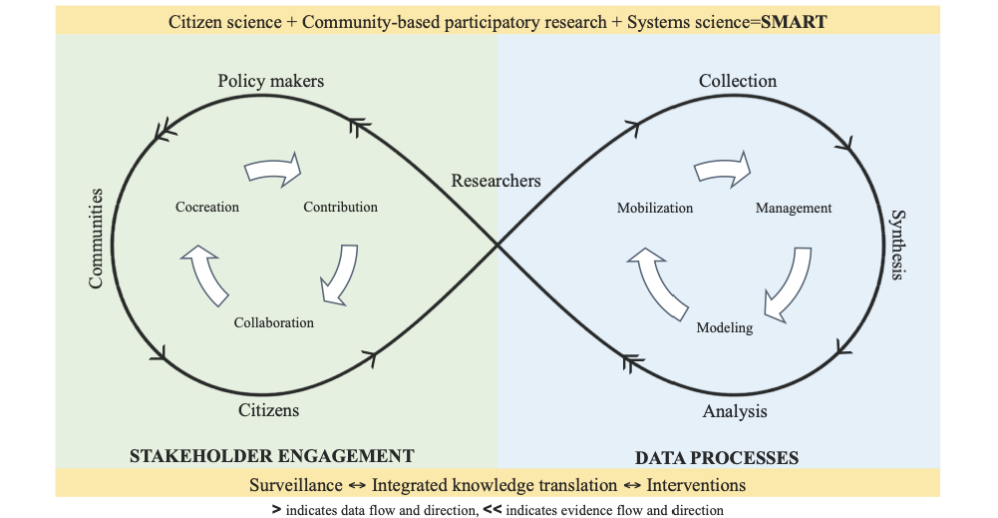
The Smart Framework.

Citizen science approaches range from contributory and collaborative methods (data collection and analysis) to cocreation of knowledge (conceptualization and knowledge translation), where all participants contribute as citizen scientists [23,27,28]. The Smart Platform is informed by an evidence-based framework that integrates citizen science, community-based participatory research, and systems science through digital tools to conduct population health research in the digital age—the Smart Framework (Figure 1) [23]. In implementing Smart Indigenous Youth, our team combines the Smart Framework with Traditional Indigenous Knowledge to ensure Two-Eyed Seeing for participatory action research [24,25]. This approach aligns with the concept of citizen science, where knowledge is cocreated with Indigenous Peoples as equal partners.

Smart Indigenous Youth is a mixed-methods 5-year longitudinal active living community trial. The initial pilot was conducted in 2019 with 2 schools located within 2 rural First Nations reserves in Saskatchewan, Canada. In Canada, Indigenous Peoples consist of 3 groups: First Nations, Inuit, and Métis. The discriminatory categorization of Indigenous Peoples in Canada is a complex subject, which is beyond the scope of this policy analysis but could be perused in Smylie and Allan’s report, “First Peoples, Second Class Treatment” [29].

A reserve is a piece of land allotted to First Nations bands under the Indian Act, where First Nations band members have the right to live, and band administrative and political structures are located. First Nations do not have title to reserve lands, which are held in trust for bands by the British Crown [30]. Reserves often consist of less valuable land and are located outside the traditional territories of First Nations. Traditional hunting and gathering, which was the livelihood of many First Nations Peoples, was severely affected by the relocation away from First Nations’ traditional territories, which were usually rich in natural resources [31].

Before the implementation of the Smart Indigenous Youth initiative, our team built strong partnerships with the communities based on equity, respect, and co-ownership. This partnership articulates not only study coconceptualization and cocreation of knowledge, but also co-ownership of data and integrated knowledge translation. Ethics approval was obtained from the Research Ethics Boards of Universities of Regina and Saskatchewan through a synchronized review protocol (REB # 2017-29).

In 2019, the study pilot year, 76 Indigenous youth citizen scientists (n=50 from School 1, n=20 from School 2), aged between 13-18 years, engaged with us in real time via a custom-built smartphone app using their own smartphones during their winter school term (ie, the intervention period). At baseline, Indigenous youth citizen scientists provided quantitative data using a combination of traditional validated measures and ecological momentary assessments via their smartphones. This quantitative data captured their physical activity, sedentary behavior, mental health, and substance abuse, among other behaviors and outcomes.

**Figure 2:**
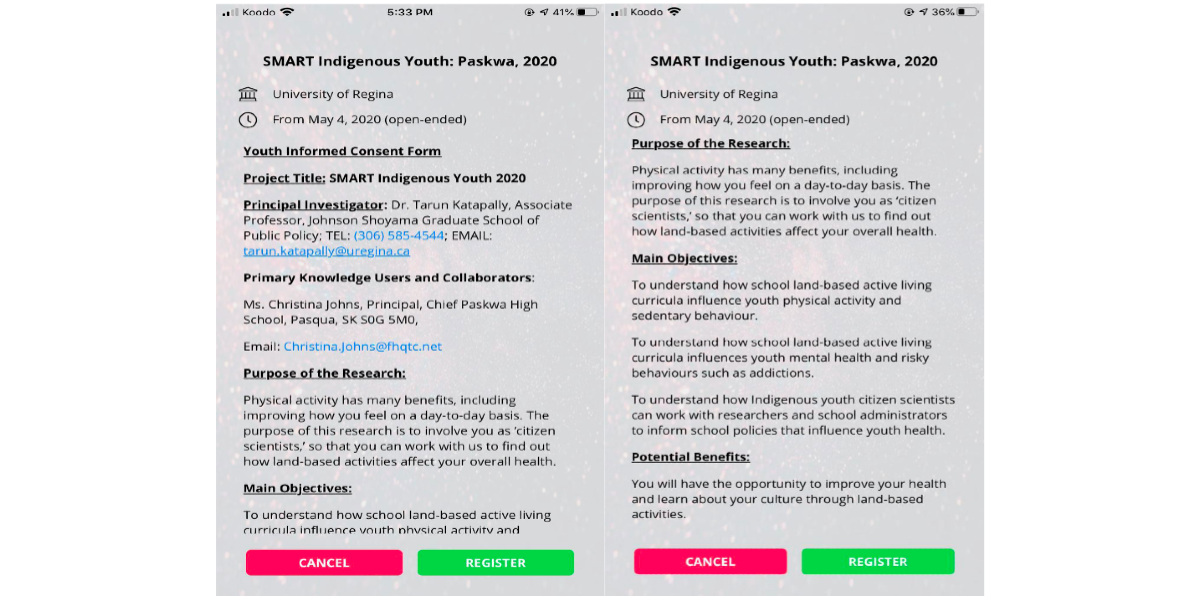
Citizen scientist informed consent via the smartphone app.

**Figure 3:**
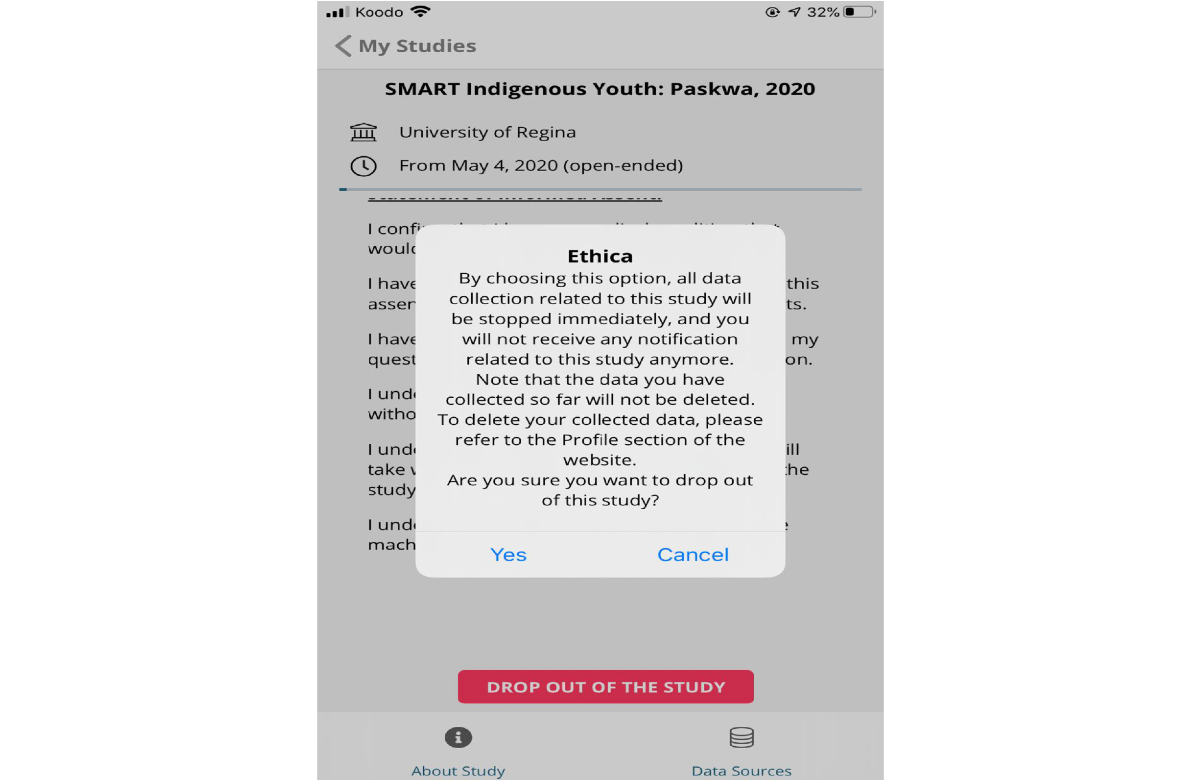
Study dropout option for citizen scientists.

**Figure 4:**
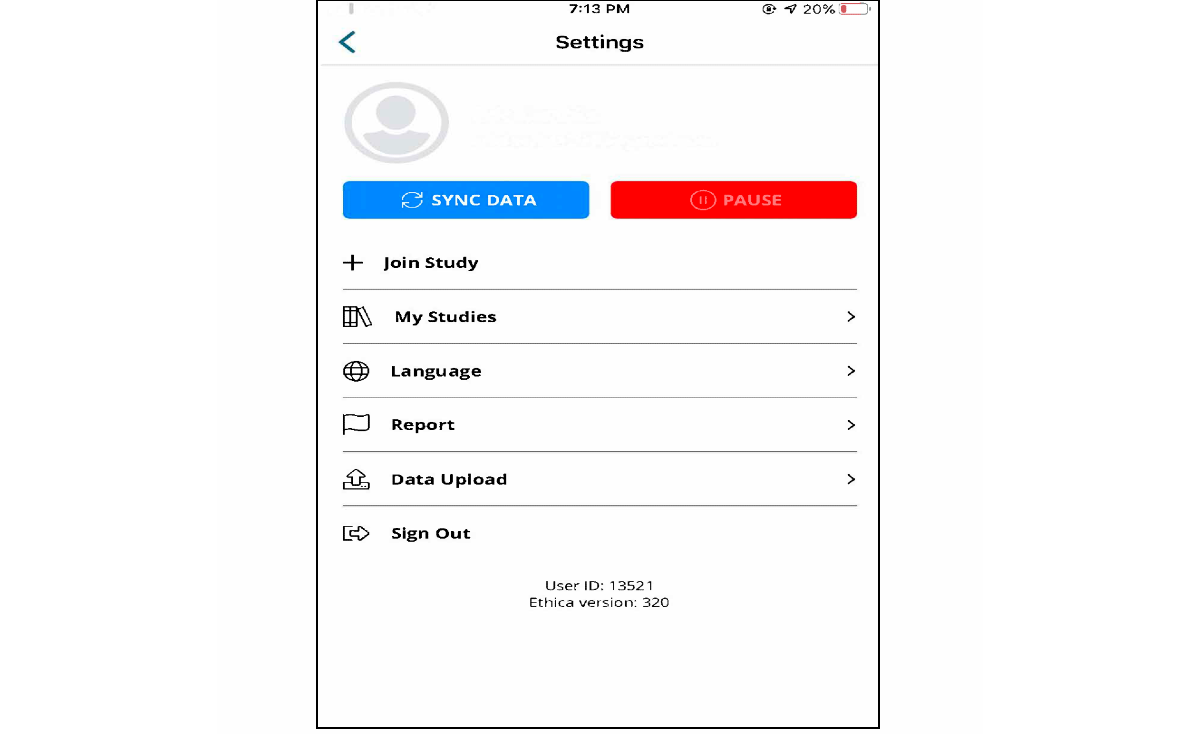
Data pause option for citizen scientists.

Before any engagement and data collection, Indigenous youth provided informed consent via the app (Figure 2) and had the option to drop out of the study (Figure 3) or pause data collection (Figure 4) at any time they wished. From the original cohort of 76 youth, 34 youth citizen scientists (n=16 from School 1, n=18 from School 2) became part of the Youth Citizen Scientist Council, which participated in baseline focus groups. Moreover, 2 school principals became educator citizen scientists to provide data on school policies and programs using the same custom-built smartphone app.

**Figure 5:**
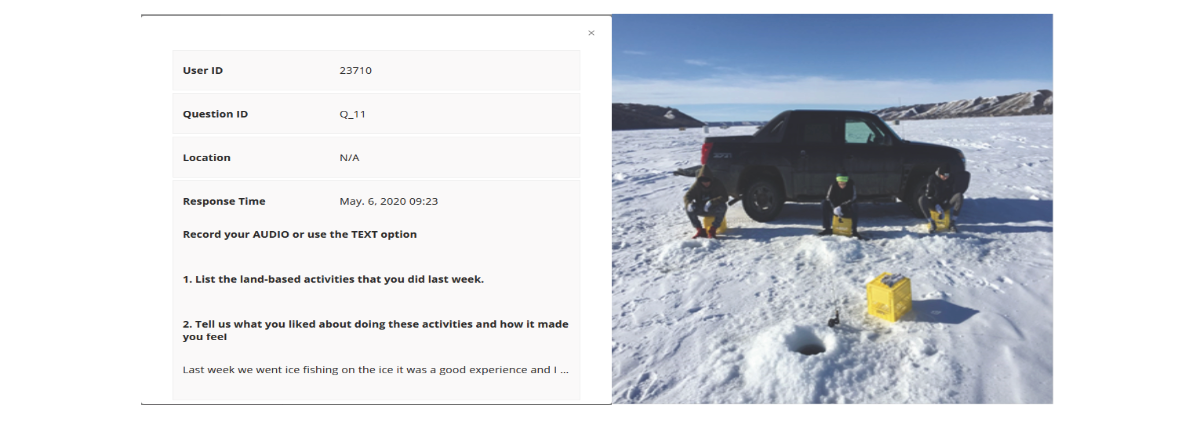
Ecological momentary assessments enabling citizen reporting of culturally appropriate land-based activities.

Thereafter, each school initiated separate 4-month (winter term) land-based active living programs that were specific to their culture, community, geography, and language (Cree and Soto). Land-based activities included traditional hunting, trapping, fishing, foraging, and plant identification, as well as recreational activities such as canoeing and hiking. After the winter term, follow-up focus groups were conducted with the Youth Citizen Scientist Council to evaluate the impact of the culturally appropriate land-based intervention. Moreover, during the 4-month intervention period, youth and educator citizen scientists engaged with researchers in real time using their smartphones to capture the perception and impact of land-based activities through time and user-triggered ecological momentary assessments (Figure 5).

The initial findings from the analysis of mixed-methods data depict the overarching importance of culture, identity, history, and language, where land-based activities such as canoeing and setting traps played a role in improved youth mental health by providing youth a sense of purpose and identity. More importantly, Indigenous youth citizen scientists continue to play a critical part in integrated knowledge translation, as evidenced by our first round of knowledge mobilization, which provides a voice to Indigenous youth to contribute to the systems integration of the Smart Platform through digital engagement [32].

## Benefits

### Digital Health Interventions Enabling Systems Integration

The primary significance of the Smart Indigenous Youth initiative is systems integration (health, education, and sport) to develop health policies that are embedded into school curricula. Current evidence clearly indicates that although Indigenous youth in rural and remote regions across the globe are more susceptible to poor mental health outcomes, existing healthcare policies do not meet the needs of Indigenous youth [33,34]. The World Health Organization’s Mental Health Action Plan 2013–2020 has a global target of a 10% reduction in suicides by 2020 by strengthening information systems [35]. It is not evident if we are closer to achieving this goal, but addressing youth mental health—especially challenges faced by Indigenous youth—should be part of long-term plans to improve global mental health.

A recent systematic review that evaluated the evidence of intersectoral interventions addressing Indigenous child and youth mental health not only recommended novel responses, but also indicated that the success of these interventions will depend on the collaboration, cultural sensitivity, and empowerment of participants [36]. Moreover, this review recommends further research on the impact on social determinants of health, the extent of participant engagement, and Indigenous voices.

Smart Indigenous Youth enables an intersectoral intervention by bringing together health, education, and sports-related policies through a culturally sensitive and land-based initiative that empowers Indigenous youth by providing them a voice. The key to the success of Smart Indigenous Youth’s systems integration is the innovation of combining citizen science, community-based participatory research, and systems science through ubiquitous devices [23]. This integration facilitates digital health interventions for mental health equity among Indigenous youth. The participatory approach of Smart Indigenous Youth creates opportunities for scalable and replicable digital health interventions since it is based on the implementation of a global digital citizen science policy [37].

### Holistic Health Benefits, Including Mental Health

Evidence is well-established that active living not only combats the effects of chronic diseases but also interconnects and balances the 4 aspects of holistic health, recognized as physical, mental, social/emotional, and spiritual/cultural, within Indigenous worldviews [38-40]. The existing mainstream structure of active living access in schools, where the focus is on competitive sports, leads to cultures of privilege, exclusivity, and isolation [39]. Such an approach to physical activity goes against established evidence that the emphasis of healthy, active living should go beyond participation in structured competitive sports [41].

Land-based active living initiatives are a conduit for connecting with cultural roots to counter the impact of historical colonization and for providing holistic healing mechanisms that facilitate Indigenous youth mental health [42]. Smart Indigenous Youth is the first initiative to utilize digital citizen science approaches to focus on culture and local Indigenous Knowledge in understanding the pathways through which land-based active living influences mental health. This initiative builds on previous research on Indigenous youth engagement with nature using photovoice and the Two-Eyed Seeing framework [43]. This nature-based research concluded that urban Indigenous youth found nature to be a calming place that enables them to cope with stress, anger, and fear, among other difficulties they face in everyday life.

### Indigenous Youth Empowerment

Given the burden of historical trauma and continued marginalization that affects the well-being of Indigenous youth, Smart Indigenous Youth uses a strengths-based approach that shifts the perceived deficits away from the individual. Instead, the emphasis is on placing health problems in the appropriate context, such as the oppression of residential schools [44]. Becoming citizen scientists allows youth to focus on their strengths and build resilience to face challenges related to anxiety, depression, and even substance abuse. Smart Indigenous Youth expands this approach to rural and remote Indigenous youth who are currently underserved by existing healthcare systems [33,34]. In implementing Smart Indigenous Youth, we focus on holistic health benefits by empowering youth with digital tools, where they engage with researchers in real time.

A key component of facilitating empowerment is building capacity among Indigenous youth for integrated knowledge translation. Smart Indigenous Youth is based on the Smart Framework’s integrated knowledge translation approach [23], which is strengthened by the Youth Citizen Scientist Council with representation from varied genders and socioeconomic groups. The evidence that is generated by this initiative is disseminated and translated in collaboration with the Youth Citizen Scientist Council, which provides a voice to Indigenous youth as depicted by our first round of knowledge mobilization [32].

## Barriers to Implementation

The primary barriers to implementation of Smart Indigenous Youth include systemic issues such as school principal employment term limits, participant burden in terms of citizen scientist compliance, and technological constraints such as internet inequity.

### Systemic Issues: Limits on School Leadership

In implementing this complex digital health initiative into school curricula, a key component is relationship building with school leadership. The school principals who co-lead this initiative are the community champions that drive the implementation along with the research lead. Without the establishment of a strong partnership with school principals, this initiative would not succeed. Moreover, building partnerships takes time, as the research lead needs to understand not only the challenges of Indigenous youth but also the historical, cultural, and socioeconomic context of the communities within which the schools exist.

The school principals ensure the successful implementation of the initiative by coordinating and integrating the initiative into existing school protocols. Thus, the current term limit of 1 year for school principals places a major barrier in implementation as there is a risk that existing school principals, with whom strong partnerships have been established, will be replaced with new leadership. Such a scenario will not only result in the loss of valuable partnerships but also slow down the continuity of implementation, as new relationships would have to be established with new principals.

### Participant Burden: The Role of Citizen Scientists

Indigenous youth and educators in each participating school participate and contribute to the Smart Indigenous Youth initiative as citizen scientists. This can cause substantial participant burden, as the expectation of citizen scientists is to play an equitable role in the initiative. This scenario varies significantly from studies where the expectation from participants ends with the provision of data. Citizen scientists in the Smart Indigenous Youth initiative have to longitudinally engage with the research team to provide not only quantitative and qualitative data using their own mobile devices but also participate in consultations for implementation and knowledge translation. As such, participation requires considerable commitment and investment in terms of time and energy, and could thus lead to considerable participant burden.

### Technological Constraints: Internet Inequity

The most significant barrier to digital health interventions, especially in rural and remote communities, is internet inequity. Internet inequity is defined as differential internet access based on wealth, location (urban, rural, or remote), gender, age, or ethnicity [23]. In our experience of working with rural and remote schools, our team has observed that although more than 90% of youth and educators own smartphones, access to Wi-Fi or cellular data plans varies widely between communities, and it is not always the case that remote communities have lower access. For example, the most remote school in our initiative provided better Wi-Fi access to their students as part of their school curricula. However, it cannot be assumed that students from more remote schools will have better access to Wi-Fi outside of school hours, especially in their homes. That is, individual socioeconomic status plays a major role in internet access outside of school hours.

## Evidence-Based Strategies to Address Barriers

### Integrating Citizen Science and Community-Based Participatory Research Action

To circumvent school principal term limits and minimize citizen scientist burden, we employ the integration of citizen science and community-based participatory research action. As citizen science can range from contributory (data collection) and collaborative approaches (analysis and interpretation of data) to cocreation of knowledge (conceptualizing research and translating knowledge) [23,27,28], it has a natural overlap with community-based participatory research action. Moreover, as community-based participatory research action is entrenched in human rights and social justice, it can be applied to promote local policy change by bringing together community needs, scientific evidence, and political power [45,46]. By taking this approach, and by turning the challenge of school principals’ 1-year terms into an opportunity, we are working to potentially expand Smart Indigenous Youth to new schools to which principals may be transferred. Thus far, we have been able to successfully expand the initiative to 1 more rural school in 2020 due to the transfer of a school principal from the 2019 pilot.

To tackle citizen scientist burden, we established the Youth Citizen Scientist Council, which provides a voice to Indigenous youth to inform and influence school policies that are relevant to their holistic health [32]. This has maximized compliance as we have structured citizen science endeavors using community-based participatory research action principles, where citizens co-design studies and cocreate knowledge with researchers by contributing to all aspects of the research process. The integration of citizen science and community-based participatory research action is being catalyzed by the ubiquitous presence of smartphones that we are leveraging to overcome traditional constraints in terms of participant recruitment and retention, data collection and analysis, interventions, and knowledge translation [27].

### Indigenization of Academia

In implementing initiatives such as Smart Indigenous Youth, strategic support from academic institutions, where the research is based, is critical for the ultimate success of these collaborative projects that are intensive in terms of human resources, time, and logistics. For instance, engaging Indigenous youth and educator citizen scientists requires recognizing their contribution with culturally appropriate incentives. This research is being conducted with a federally funded grant that earmarks participant incentives in the budget; however, there was a need to indigenize the financial processes to ensure the timely delivery of incentives.

For instance, our team had to influence purchasing and reimbursement processes so that citizen scientists received incentives before they participated in the initiative. Similarly, we also had to influence financial regulations to ensure that the relevance of incentivizing Indigenous citizen scientists is not questioned at the institutional level. Ultimately, the success of this difficult bureaucratic task depended on academic leadership in engaging citizens, communities, and institutional management to address population health issues that are of concern to citizens, communities, policymakers, and researchers.

### Local Solutions to Tackle Internet Inequity

As part of the Smart Indigenous Youth initiative, we are engaging with policymakers, communities, schools, and citizens to develop bottom-up approaches for improving internet access to Indigenous youth. We are addressing this complex issue at 2 levels: (1) by engaging schools to provide participating youth access to school Wi-Fi before, during (class breaks), and after school hours; (2) by budgeting funds to provide mobile data plans to the most disadvantaged youth. In our experience, individual data plans are better suited than mobile Wi-Fi hotspots because they provide continuous data access to enable data capture in free-living conditions, without changing behavior patterns.

## Conclusion

Digital health interventions have a tremendous potential to bridge geographic, economic, and social disparities by leveraging ubiquitous tools that are accessible to Indigenous youth in rural and remote communities of the world. The Smart Indigenous Youth initiative embeds a digital health intervention into school curricula to integrate policies across systems. Moreover, this initiative empowers youth through culturally appropriate, land-based active living that is critical for their mental health. The Smart Indigenous Youth initiative provides a scalable and replicable model for digital health approaches that could be particularly effective in eventually developing policies to address the risk of mental illness in real time.
